# Diet diversity and environment determine the intestinal microbiome and bacterial pathogen load of fire salamanders

**DOI:** 10.1038/s41598-021-98995-6

**Published:** 2021-10-14

**Authors:** Yu Wang, Hannah K. Smith, Evy Goossens, Lionel Hertzog, Molly C. Bletz, Dries Bonte, Kris Verheyen, Luc Lens, Miguel Vences, Frank Pasmans, An Martel

**Affiliations:** 1 Wildlife Health Ghent, Department of Pathology, Bacteriology & Avian Diseases, Ghent University, Salisburylaan 133, 9820 Merelbeke, Belgium; 2 Department of Pathology, Bacteriology & Avian Diseases, Ghent University, Salisburylaan 133, 9820 Merelbeke, Belgium; 3 Terrestrial Ecology Unit (TEREC), Department of Biology, Ghent University, K. L. Ledeganckstraat 35, 9000 Ghent, Belgium; 4 Thünen Institute for Biodiversity, Bundesallee 68, 38116 Brunswick, Germany; 5 Evolutionary Biology Lab, Zoological Institute, Braunschweig University of Technology, Mendelssohnstr. 4, 38106 Brunswick, Germany; 6 Forest & Nature Lab, Department of Environment, Ghent University, Geraardsberge Steenweg 267, 9090 Gontrode, Belgium

**Keywords:** Ecology, Biodiversity

## Abstract

Diverse communities of symbiotic microbes inhabit the digestive systems of vertebrates and play a crucial role in animal health, and host diet plays a major role in shaping the composition and diversity of these communities. Here, we characterized diet and gut microbiome of fire salamander populations from three Belgian forests. We carried out DNA metabarcoding on fecal samples, targeting eukaryotic 18S rRNA of potential dietary prey items, and bacterial 16S rRNA of the concomitant gut microbiome. Our results demonstrated an abundance of soft-bodied prey in the diet of fire salamanders, and a significant difference in the diet composition between males and females. This sex-dependent effect on diet was also reflected in the gut microbiome diversity, which is higher in males than female animals. Proximity to human activities was associated with increased intestinal pathogen loads. Collectively, the data supports a relationship between diet, environment and intestinal microbiome in fire salamanders, with potential health implications.

## Introduction

The digestive system of vertebrates is home to a dynamic microbiome^1,2^, which forms a ubiquitous and complex symbiotic relationship with their host. This symbiosis has long been known to support the production of vitamins^3^, the chemical processing of indigestible chyme components by bacterial fermentation^4^, and degrading toxic substances^5^. Moreover, the gut microbiome was also found to play a role in the maturation of the immune system^6^ and even ecological adaptation^7^. The composition of the gut microbiome differs greatly among host taxa, as well as the primary factors determining it. For termites^8^ and non-human primates^9^, the gut microbiome is strongly associated with host physiology and phylogeny. For fish^10,11^, birds^12,13^, myrmecophagous mammals^14^, mice^15^ and amphibians^16,17^, host diet has been found to be one of the primary determinants in shaping microbial communities. Other factors, such as climate^18^, habitat^19,20^, and the host immune system^21^ also drive internal microbiome diversity of certain animal clades to some extent. Host diet not only serves as a source of potential gut colonists^22^, but also modulates gut microbiome dynamics^23^. Empirical studies on gut microbiomes and its relation to diet in additional species have the potential to further shed light on this delicate ecological balance.

Amphibians are among the world’s most vulnerable groups of animals, with 40% of species in danger of extinction^24–26^ due to habitat destruction, climate change and emerging diseases^27–32^. Across the globe, amphibians are a key part of many ecosystems^33,34^, making up a large proportion of vertebrate communities in forest, tropical and wetland ecosystems both in terms of individual abundance and overall biomass^35–37^. Frogs and salamanders influence leaf litter decomposition as well as nutrient cycling, by preying upon omnivore and detritivore invertebrate populations^38–41^. This also applies to fire salamanders (*Salamandra salamandra*), a species occurring throughout most of temperate deciduous forests in central and southern Europe^42^. They can occur in high densities and are deeply woven into local food webs, contributing to ecosystem stability^40^. Understanding such food web data is important for drafting conservation strategies and understanding community ecology and ecosystem functioning^31,43,44^.

In this study, we use DNA metabarcoding to profile both the diet and intestinal bacterial composition of fire salamander populations in Belgian forests. The aim is to provide detailed information on the diversity, relative abundance and prevalence of prey taxa found within fecal samples of fire salamanders, as well as the impact of diet, sex and geographical location on the gut microbiome.

## Results

### Diet analysis based on fecal DNA metabarcoding

A total of 20 prey taxa were identified, belonging to the phyla Mollusca, Annelida and Arthropoda.

**Figure 1:**
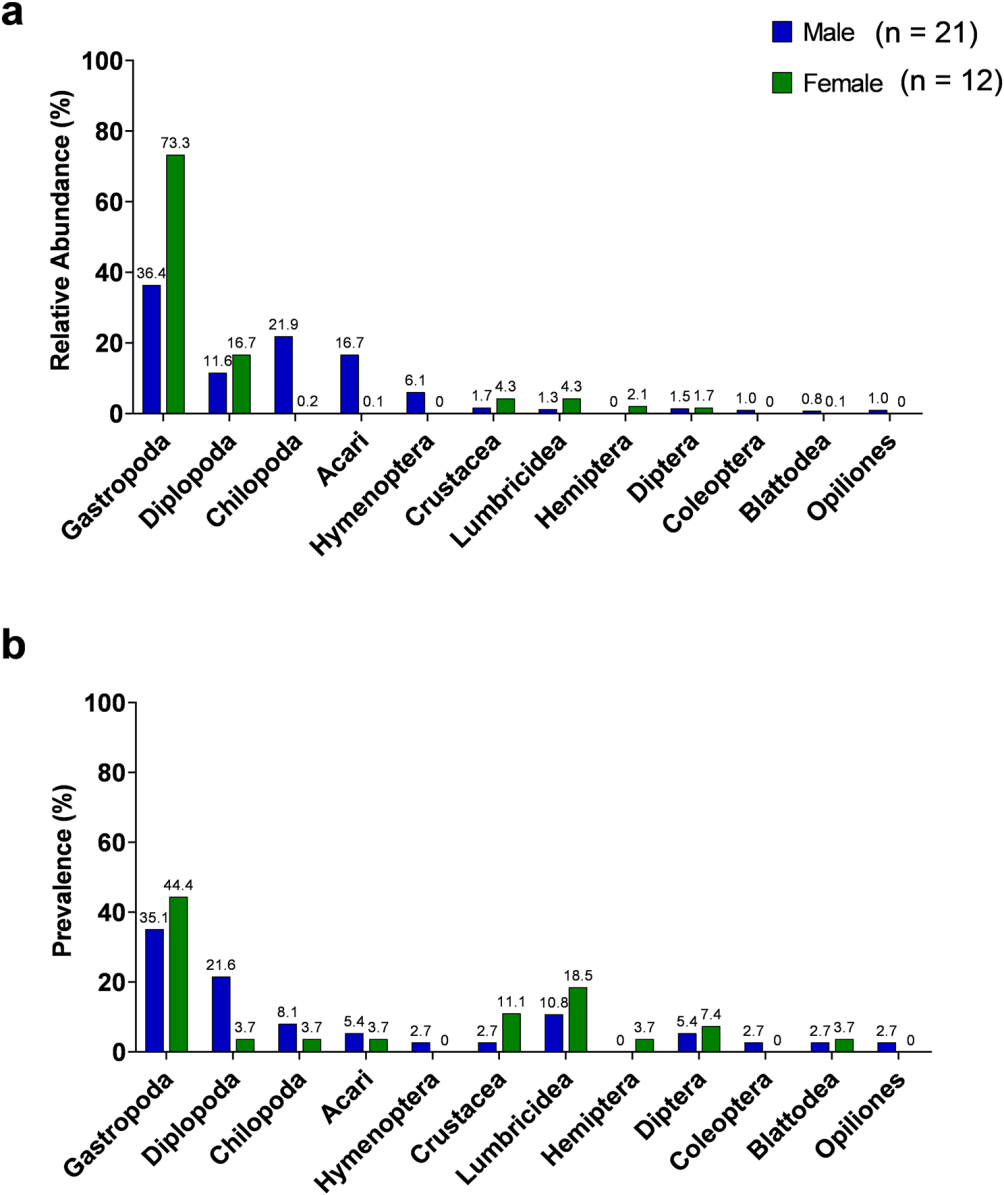
Diet composition between sexes. (**a**) Relative abundance of fire salamander diet in male and female animals. Relative abundance: sub-OTU reads of each prey category to the amount of reads assigned to total prey. (**b**) Prevalence of prey taxa in male and female animals. Prevalence: the number of samples in which a prey category was found (presence/absence).

Fire salamander ingested prey biomass was determined by the relative number of sub-OTU reads for each prey category. We used this as a proxy for the relative amount of ingested prey within all salamander fecal samples. More than 70% of the sub-OTU reads were identified as gastropods, followed by millipedes at 10.8%, centipedes at 5.8%, and soil mites at 4.4%. All other taxa were rare in fire salamander diet. Diet taxa prevalence (presence/absence) across all salamanders revealed gastropods to be the most prevalent taxonomic class at 40.6%. Both millipedes and annelids (earth worms) were found at 14.5%. Centipedes, true flies and crustaceans made up 5.8% of salamander diet. Relative abundance of prey sub-OTUs differed significantly between females (n = 12) and males (n = 21) (RDA F = 4.58, df = 2.28, *p* = 0.011). Prey ingested by females was made up largely of gastropods (73.3%), with millipedes making up most of the remaining prey (16.7%). Males, on the other hand, had a more balanced diet, with a large portion of their ingested prey being divided between gastropods (36.4%), millipedes (21.9%), soil mites (16.7%), and centipedes (11.6%) (Fig. 1a). Prevalence of prey taxa also differed significantly between both sexes (χ^2^_1,12_ = 35.12, *p* < 0.001) (Fig. 1b).

Fire salamander diets were largely made up of the same general taxa, and the relative abundances of prey reads did not differ between the forests of HG (Heilig Geestgoed) (total n = 11, male n = 5, female n = 5, juvenile n = 1), M (Makegem) (total n = 17, male n = 9, female n = 5, juvenile n = 3) and S (Smetledebos) (total n = 9, male n = 7, female n = 2, juvenile n = 0) (RDA F = 0.50, df = 2.28, *p* = 0.738). Gastropods and earthworms were found to have a similar prevalence in salamander diets between forests (Supplementary Figure S1). Differences were seen in millipedes, where values fluctuated between locations, although not significantly (χ^2^_2,12_ = 4.35, *p* = 0.113). Many of the Insecta taxa were not part of the salamander diet in all three forests (Supplementary Figure S1).

**Figure 2:**
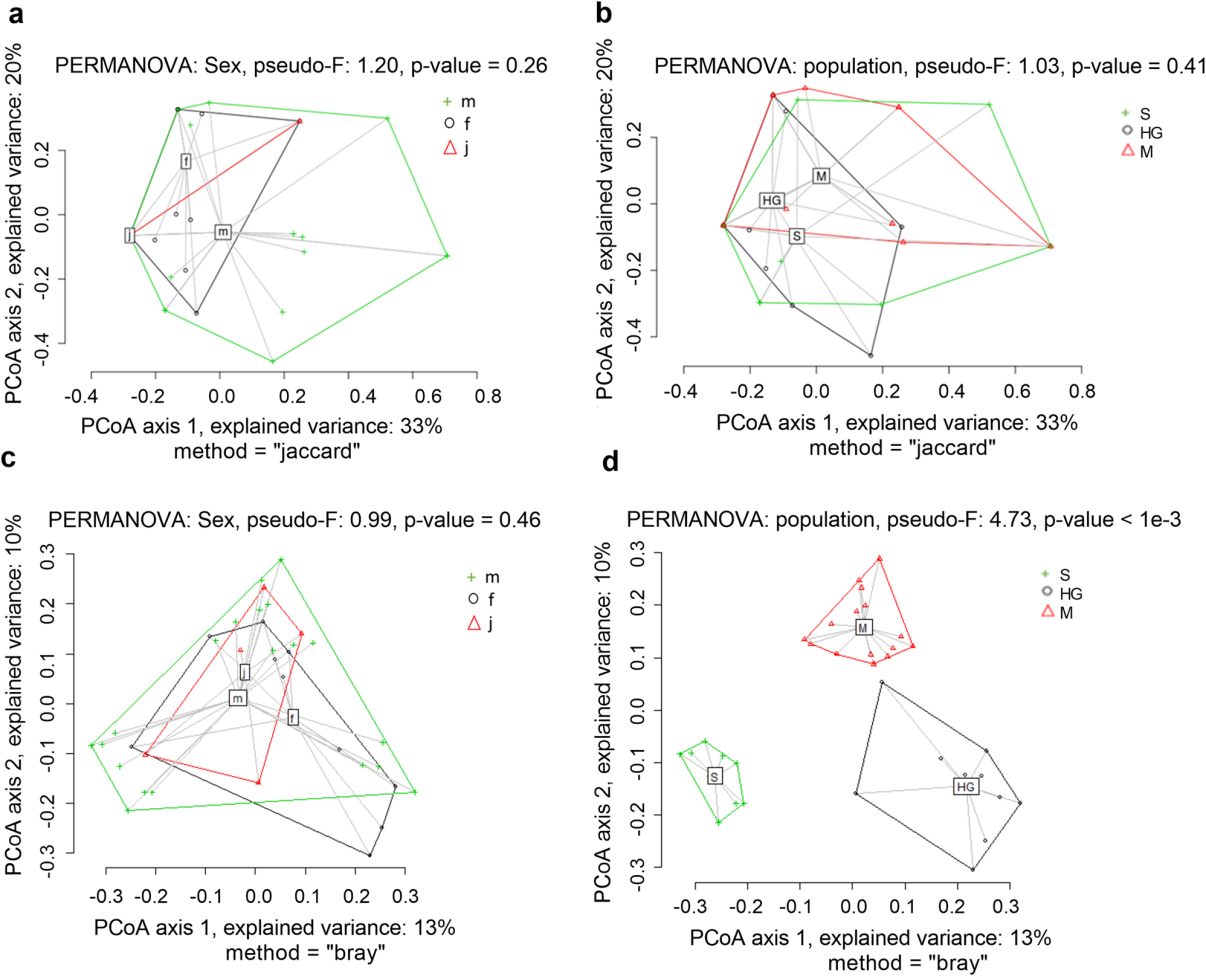
Principal coordinates analysis of Jaccard beta diversity matrices for diet of sexes (**a**) and locations (**b**). Principal coordinates analysis based on Bray–Curtis dissimilarity distance for microbiome composition of sexes (**c**) and locations (**d**). m = male, f = female, j = juvenile, HG = forest Heilig Geestgoed, M = forest Makegem and S = forest Smetledebos.

Alpha diversity of the salamander diet did not differ between locations (Chao2 H_2_ = 0.013, *p* = 0.994; sub-OTU richness H_2_ = 0.016, *p* = 0.992) or sexes (Chao2 W = 99, *p* = 0.246; OTU Richness W = 108, *p* = 0.407). Beta diversity of the salamander diet showed a large overlap between sexes, as well as forests (Fig. 2a,b) and there was no significant difference in diet composition between sexes (PERMANOVA Pseudo-*F* = 1.20, df = 2. 30, *p* = 0.26), as well as locations (PERMANOVA Pseudo-*F* = 1.03, df = 2. 30, *p* = 0.41). Moreover, we found that the salamander body condition (SMI) did not correlate to the diversity of diet (OTU richness r_s_ = 0.074, *p* = 0.640; Chao2 r_s_ = 0.067, *p* = 0.694).

### Gut microbiome analysis and pathogen load based on fecal DNA metabarcoding

Within all fecal samples, we found 15 different bacterial phyla. Bacteroidetes made up the largest portion of bacteria at 47.8%, followed by Firmicutes at 32.1% and Proteobacteria at 15.3%. These three phyla made up 95.2% of all bacteria found in fecal samples. The remaining bacteria were identified as Verrucomicrobia (3.7%), Desulfobacterota (0.6%), Cyanobacteria (0.2%), Actinobacteriota (0.1%), Elusimicrobiota (0.1%), Deferribacterota (0.009%), Bdellovibrionota (0.006%), Patescibacteria (0.003%), Myxococcota (0.001%), Planctomycetota (0.001%), Fusobacteriota (0.001%), and others (0.001%). When comparing the fecal microbiome from salamanders captured in different forests, significantly more Proteobacteria and Elusimicrobia were observed in forest HG, as compared to forest M or S (Supplementary Table S1).

**Figure 3:**
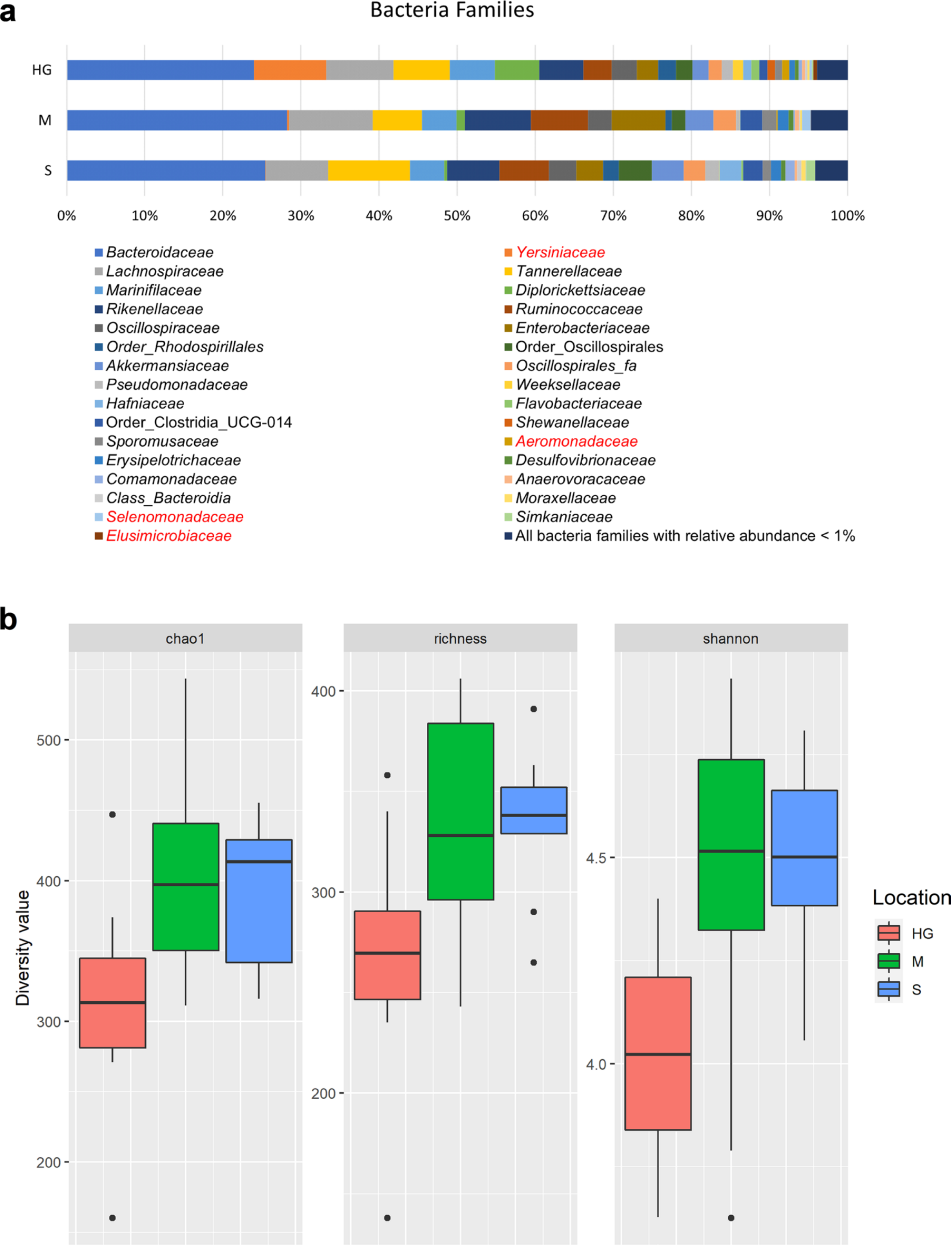
(**a**) Bacteria families found in the fecal samples of fire salamanders from forests HG (Heilig Geestgoed), M (Makegem), and S (Smetledebos). Bacteria families that are significantly different between locations are highlighted in red. (**b**) Alpha diversity indices of gut microbiome between different locations. Boxes are extended from the 25th to 75th percentiles, and the horizontal line inside the boxes defines the median. Whiskers indicate variability outside the upper and lower quartiles. Black circle indicates outlier.

Looking at the family level, Bacteroidetes was represented by four main families: *Bacteroidaceae* (HG 24.0%/M 28.2%/S 25.4%), *Tannerellaceae* (HG 7.2%/M 6.3%/S 10.5%), *Rikenellaceae* (HG 5.7%/M 8.5%/S 6.7%) and *Marinifilaceae* (HG 5.7%/M 4.4%/S 4.4%). For Firmicutes the four main families were: *Lachnospiraceae* (HG 8.6%/M 10.7%/S 7.9%), *Ruminococcaceae* (HG 3.6%/M 7.3%/S 6.4%), *Oscillospiraceae* (HG 3.2%/M 3.1%/S 3.5%) and *Oscillospirales_fa* (HG 1.7%/M 2.9%/S 2.7%). Proteobacteria was represented by: *Yersiniaceae* (HG 9.3%/M 0.2%/S 0.1%), *Enterobacteriaceae* (HG 2.8%/M 6.8%/S 3.4%), *Diplorickettsiaceae* (HG 5.7%/M 1.1%/S 0.4%) and *Pseudomonadaceae* (HG 1.4%/M 0.5%/S 1.8%). Most of the remaining families within these phyla contain less than 1% of the relative abundance (Fig. 3a). In total, 138 families were found to be present in at least one location. Of those, only four families were identified to be significantly different in abundance between locations (Supplementary Table S1).

Based on redundancy analysis (RDA), the gut microbiome composition was significantly affected by location (RDA *F*_2,36_ = 4.07, *p* = 0.001) but not by sex (RDA *F*_*2,36*_ = 0.85, *p* = 0.720). Alpha diversity of the gut microbiome varies between locations (Fig. 3b), with significant differences for ASV richness (χ^2^ = 7.52, df = 2, *p* = 0.023), Chao1 (χ^2^ = 9.18, df = 2, *p* = 0.010) and Shannon Index (χ^2^ = 11.09, df = 2, *p* = 0.004). We found that forest HG has significant lower alpha diversity than the other two forests (Fig. 3b). When comparing between sexes, alpha diversity of the gut microbiome is significantly lower for females than males: ASV richness (W = 43, *p* = 0.013), Chao1 (W = 48, *p* = 0.021), Shannon Index (W = 38, *p* = 0.005). The dissimilarity in microbiome composition was analyzed with PERMANOVA on Bray–Curtis distance, where no difference could be seen comparing between sexes (Fig. 2c, Pseudo-*F*_2,29_ = 0.9, *p* = 0.556), but when comparing between locations, the three forests were significantly different from each other (Fig. 2d, Pseudo-*F*_2,29_ = 4.73, *p* < 0.001). Moreover, SMI (RDA *F*_*1,19*_ = 1.80, *p* = 0.340) was not correlated with gut microbiome composition.

We included pathogens of the genera *Flavobacterium*, *Chryseobacterium*, *Sphingobacterium*, *Aeromonas*, *Citrobacter*, *Yersinia*, *Acinetobacter* and *Stenotrophomonas* as a proxy for pathogen load, given their known involvement in amphibian pathology^45–55^. The presence of potential pathogenic amphibian bacteria in the fire salamander gut microbiome was analysed at genus level (Supplementary Figure S2). We focused on eight genera well known to contain pathogenic bacterial taxa (Table 1). The total relative abundance of the eight pathogenic bacterial genera makes up less than two percent of the fire salamander gut microbiomal communities in forest M (0.5%) and S (1.4%), but more than 13% in forest HG. The relative abundance of the pathogen load is significantly higher in forest HG, compared to forest M (*p* < 0.0001). Similarly, a tendency towards increased pathogen load was observed when comparing forests HG and S (*p* = 0.8649). Comparison of beta diversity of the pathogen load between forests was analysed with PERMANOVA on Bray–Curtis distance and revealed a significant difference between locations (Supplementary Figure S3, Pseudo-F = 4.69, df = 2. 80, *p* = 0.0001).

**Table 1:** Mean relative abundance and level of significance of potentially pathogenic bacterial genera in the fire salamander gut microbiome between different forests (HG = forest Heilig Geestgoed, M = forest Makegem and S = forest Smetledebos).

Genus	Mean relative abundance			*p*-value		
	HG (%)	M (%)	S (%)	HG-M	HG-S	M-S
*Flavobacterium*	1.0	0.02	0.3	0.976	0.550	0.898
*Chryseobacterium*	1.3	0.01	0.1	0.669	1.000	1.000
*Sphingobacterium*	0.1	0.002	0.004	1.000	1.000	1.000
*Aeromonas*	0.9	0.1	0.0	0.614	< 0.001	< 0.001
*Citrobacter*	0.2	0.1	0.3	0.573	0.882	1.000
*Yersinia*	9.2	0.02	0.05	< 0.001	< 0.001	1.000
*Acinetobacter*	0.3	0.2	0.6	0.809	0.967	1.000
*Stenotrophomonas*	0.1	0.01	0.04	0.951	1.000	1.000

### Diet and gut microbiome

A Mantel test revealed that dietary differences between individual salamanders are not similar to the microbiome differences (r = − 0.05, *p* = 0.747). Exploring the structure between diet and microbiome we found a co-inertia coefficient of 0.42, indicating that the two categories varied independently (*p* = 0.721). Furthermore, no correlation was observed between the alpha diversity (Chao1 index) of diet and gut microbiome (Supplementary Figure S4, Spearman’s r_s_ = − 0.05, *p* = 0.748).

## Discussion

To estimate the accuracy of the DNA barcoding technique, validation studies have been conducted using feeding experiments of captive animals, where exact diet inputs were compared to fecal DNA sequencing outputs^56–58^. Whilst the species of prey could be successfully identified in these studies, the proportion of detected DNA varied and was not completely on par with the dietary proportions. Amounts of DNA in the fecal matter need to be exactly proportionate to ingested prey biomass, which is not always the case, due to biological and technical biases, such as different speeds of digestion, size of ingested prey, possible presence of multicopy genes, DNA degradation in fecal samples and availability of DNA reference sequences of potential prey in public databases^59–61^. Nonetheless, DNA sequencing of fecal matter is a more viable technique to identify soft-tissued, easily digestible prey^59^, and able to provide higher resolution than conventional stomach content analysis^62,63^. This technique has been applied successfully in unravelling diets for many species, including birds^44,56^, mammals^59,64,65^, fish^66,67^ and reptiles^68^, but so far has not been used for amphibians.

In this study, we characterise the diet of fire salamanders by means of DNA metabarcoding of fecal samples. Our data identifies gastropods as the most prevalent prey of fire salamanders, and reveals the diet differences between sexes. As reported previously, fire salamanders are opportunistic and generalist predators that focus on slow-moving and soft prey items^69–72^. Our findings are in line with previous publications of fire salamander diet compositions, where Gastropoda was also found to be the most important prey^71^. Other amphibian species have significantly different diet compositions. Monte Albo cave salamander, for example, mostly consume Hymenoptera, Ambrosi's cave salamander feed mostly on Arachnida and Strinati's cave salamander mostly consume Diplopoda^73–75^. This likely explains why our results are in line only with previous studies on diet conducted specifically on fire salamanders. The sex-dependent change in diet has been thoroughly investigated in a number of amphibian species^73,75–78^, but no difference of diet composition has been found. Our study observed for the first time diet differences between sexes in amphibians, with female fire salamanders consuming more gastropods and millipeds. While in other vertebrates, such as reptiles, this is often linked to sexual dimorphism^79–81^ and/or the utilisation of different microhabitats^82^, we attribute the observed difference to different activity patterns. In North American plethodontid salamanders it has been found that females are less active than males^83–85^, resulting in them eating more slow-moving organisms, such as millipedes and gastropods^86^. Similarly, during the breeding season in autumn, female fire salamanders are less active outside the shelters than males^82^, which could explain the diet difference between sexes.

Interestingly, the alpha diversity of the gut microbiome was also found to be significantly different between sexes, with diversity of species being lower in female than in male animals. Looking at this lower diversity of the gut microbiome in female animals, especially with regards to the sex-dependent diet, these findings suggest that decreased prey variation results in lower gut microbiome diversity. Previous studies on gut microbiome compositions between sexes in amphibians have yielded mixed results. One study on Chinese concave-eared frogs observed a significant difference in gut microbiome composition at family and genus levels between sexes^87^. In cane toads a study elucidated that the difference in variation of gut microbiome communities is mostly due to the factor of sex, but did not statistically quantify the effect on microbiome diversity^88^. In rice field frogs, however, no statistical difference of gut microbiome composition could be found between sexes^89^. Therefore, differences of gut microbiome diversity between sexes depends on the respective species, and is most likely subject to individual behaviour, prey selection, biology and environmental factors.

The alpha diversity of the fecal micriobiome was also found to be significantly different between locations, with a lower diversity of species found at forest HG, which is located adjacent to farmland, unlike the other two forests in this study. Forest HG also stood out, in that it had a remarkably higher pathogen load, compared to the other forests. We assume that the higher pathogen load coincided with the close vicinity to farmland. This hypothesis is supported by the relatively high amount of Proteobacteria, and the presence of members of the phylum Elusimicrobia in these salamanders, which has been associated with exposure to fertilizers and pesticides in farmland frogs^90^. Generally, a substantial proportion of Proteobacteria is typical for amphibian gut microbiomes, but more so in larvae than adults^17,91,92^. An additional explanation is that, in contrast to the other two sites, the salamanders from this forest are exposed to raw sewage from the neighbouring houses, which may also explain increased numbers of *Yersiniaceae*, notably of the genus *Yersinia*, which has been previously reported as an indicator of fecal pollution in fish^93^. *Yersinia* has also been reported in amphibian species, such as *Necturus maculosus*^94^, *Rana pipiens*^95^ and *Rana clamitans*^45^. This, however, is the first time *Yersinia* has been found in gut microbiome of amphibians from a farmland adjacent habitat, which may prossibly be linked to sewage and fecal pollution. Therefore, the salamander gut microbiome is likely associated with land use and/or pollution, which may have consequences for salamander physiology and health.

Furthermore, we observed no correlation between diet alpha diversity and gut microbiome alpha diversity in fire salamanders. However, previous studies on the correlation between microbiome richness and dietary richness did not yield consistent results. A positive correlation between diet diversity and gut microbome diversity was found in humans^96^, black howler monkeys^97^, kudus^98^ and predatory insects^99^. In contrast, a negative correlation was observed in fish, where a study showed that feeding on mixed diets resulted in a lower gut microbiome diversity, compared to pure diets^11^. Moreover, no correlation between diet diversity and gut microbiome diversity was observed in numerous mammalian herbivore species, such as pika, elephant and camel^98^. Diet-microbiome correlation is thus difficult to distill into a general rule across the animal kingdom. A previous study looked specifically at the diet and gut microbiome correlation, where a positive correlation was found in tadpoles of Malagasy frogs^17^. We were unable to confirm this in our study. So for now it appears that variety of diet does not always correlate with the variety and biodiversity of the gut microbiome in amphibians.

Similar studies of the gut microbiome have been conducted in fire salamander larvae^20^, where they found Proteobacteria, Firmicutes and Bacteroidetes to be the most abundant phyla. This is in line with our findings, where we have found Bacteroidetes, Firmicutes and Proteobacteria to be the most abundant phyla. Exception is the phylum Actinobacteriota, which was found to be more abundant in both the pond larvae (3.9%) and stream larvae (6.6%), than in our adult fire salamanders (0.1%). Nonetheless, these findings could indicate that the most abundant phyla in the gut microbiome composition do not undergo major changes throughout the different life stages of fire salamanders. The aforementioned publication also found that gut microbiome composition changes depending on habitat-specific diet in ponds versus streams. We were unable to see such stark contrasts of habitat-dependent diet in our adult animals, since prey was similar across different forest habitats.

To conclude, this is the first study to investigate the correlation between the diversity of diet and gut microbiome of adult fire salamanders in Belgian forests, using high-throughput DNA metabarcoding techniques. We show that diet composition is driven by sex, and influences microbiome composition in the fire salamander intestine. However, no correlation was observed between diet diversity and gut microbiome diversity.

## Methods

This study was carried out in compliance with the ARRIVE guidelines and all methods were carried out in accordance with relevant guidelines and regulations.

### Field sampling

All samples were collected from October to November 2016 at three forest locations in East Flanders, Belgium. Heilig Geestgoed (hereafter HG), a 29.77 ha public forest in the municipality of Merelbeke with 9 ponds (latitude 50.946881, longitude 3.726310). Makegem (hereafter M), a 54.24 ha private forest in Harentbeekbos with 10 ponds (latitude 50.945331, longitude 3.714886) approximately 1 km from HG and finally Smetledebos (hereafter S) with 45.75 ha, a public forest with 4 ponds (50.976308, longitude 3.906562), approximately 22 km from all other forests. All locations from which the salamanders were collected consisted predominantly of beech trees (*Fagus sylvatica*) with minimal undergrowth. Permits for sampling and experimental protocols of fire salamanders were granted by Agentschap voor Natuur en Bos of East Flanders in Belgium, license number ANB/BL/FF-V15-00015. According to the Belgian and EU legislation (EU directive 2010/63/EU), the collection of faeces obtained after spontaneous defecation is not considered an animal experiment and therefore does not require the approval of an ethical committee. Permission for collecting the samples in the forests (public and private) were obtained from the owners in the framework of the UGent GOA project Scaling up Functional Biodiversity Research: from Individuals to Landscapes and Back (TREEWEB).

### Sample collection

Fire salamanders were collected from each of these three forests. They were kept individually without food in sterile boxes (16 × 11 × 5 cm) with moist towel, air holes and hiding places for 1 to 3 days at 15 °C at the Faculty of Veterinary Medicine, University of Ghent in Merelbeke and then returned to their exact same locality in the forest. All animals were weighed (to 0.1 g) and measured (to 0.1 cm). Scaled mass index (SMI) of body condition (${\widehat{\mathrm{M}}}_{i}$) was calculated for all salamanders: ${\widehat{\mathrm{M}}}_{i}={\mathrm{M}}_{i}[\frac{{L}_{0}}{{L}_{i}}{]}^{{b}_{SMA}}$^100^. Animals were checked and the boxes were cleaned each day and any fecal samples were collected in Eppendorf tubes and frozen at − 20 °C until DNA extraction. In total, 60 fecal samples were collected from 49 individual fire salamanders. 13 individual fire salamanders were collected from forest HG (7 males, 5 females and 1 juvenile), 21 individuals from forest M (11 males, 6 females and 4 juveniles) and 15 individuals from forest S (9 males, 4 females and 2 juveniles).

### Fecal sample DNA metabarcoding

DNA was extracted from all fecal samples with the MoBio PowerSoil DNA Isolation Kit (MoBio, Carlsbad, CA, USA) following the manufacturer’s protocol. Extracted DNA was stored at − 20 °C until further processing.

### Diet

Sequencing methods were used as previously described^101^. The V9 regions of nuclear 18S rRNA gene were amplified using modified primers 1391F (5′-GTACACACCGCCCGTC-3′) and EukBr (5′-TGATCCTTCTGCAGGTTCACCTAC-3′)^102^. We performed index PCR with unique combinations of indexed forward and reverse primers for each sample using Phusion High-Fidelity DNA Polymerase (Thermo Fisher Scientific). For quantification of amplicon DNA concentration, we roughly assessed intensity of their signal on agarose gels and then added 2 to 6 µl to a pooled library. This library was gel-purified by cutting out the band of the correct amplicon size, and subsequently cleaned with Qiagen MinElute Kit. DNA concentration of the purified library was determined with a Qubit 2.0 fluorometer. The library was sequenced on the Illumina MiSeq platform using MiSeq Reagent Kit v3 for 300 cycles in both directions, including 10% phiX. Sequences were processed with MacQIIME v1.9.1^103^, filtering the forward reads as previously described^101^. Quality filtered sequences were clustered into sub-operational taxonomic units (sub-OTUs) using the deblur workflow^103,104^; (https://github.com/biocore/deblur). Within this workflow, all sequences were trimmed to 150 bp and subsampled to 13,000 reads per sample. Sub-OTUs clusters with less than 10 reads were removed. Sequences of sub-OTUs were taxonomically identified through BLAST searches. Only potential salamander prey items were selected from the 18S data, discarding sub-OTU reads from taxa such as Bacteria, Fungi and Protists. Prey taxa were identified into the main taxa: Gastropoda, Diplopoda, Chilopoda, Arachnida (Acari and Opiliones), Crustacea, Annelida (Lumbricidae), and six insect clades: Hymenoptera, Hemiptera, Dipera, Coleoptera, Blattodea and Collembola (Supplementary Table S2).

### Gut microbiome

The V3-V4 hypervariable region of the 16S rRNA gene was amplified using gene-specific primers S-D-Bact-0341-b-S-17 and S-D-Bact-0785-a-A-21^105^. The PCR amplification procedures were performed according to a previous study^106^. The final barcoded libraries were combined to an equimolar 10 nM pool and sequenced with 30% PhiX spike-in using the Illumina MiSeq v3 technology (2 × 300 bp, paired-end) at the Oklahoma Medical Research Centre (Oklahoma City, USA). Demultiplexing of the amplicon dataset and deletion of the barcodes was done by the sequencing provider. Quality of the raw sequence data was checked with the FastQC quality-control tool (Babraham Bioinformatics, Cambridge, United Kingdom; http://www.bioinformatics.babraham.ac.uk/projects/fastqc/), after which the sequences were trimmed, quality-filtered and dereplicated using the DADA2 algorithm (v1.14) within R^107^. An initial amplicon sequence variant (ASV) table was constructed before chimaeras were identified using the *removeBimeraDenovo* function. Finally, taxonomy was assigned using DADA2’s native naïve Bayesian classifier against the Silva database (v138)^108^.

To select the appropriate subsampling depth, alpha rarefaction curves were generated (Supplementary Figure S5). One sample (sample MF12) was unsufficiently sequenced, and was therefore excluded from the final ASV table. Amplicon sequence variants were subsampled (rarefied) by random sampling to a depth of 5047 ASVs per samples (depth of the least sequenced sample).

As our sequencing samples are high bacterial biomass faecal samples, negative controls (buffer control) were included in our sequencing runs, to guard against reagent contamination, as this is only a problem in low bacterial biomass samples. We rarefied to 1300 reads per sample for 18S and 5047 ASVs for 16S and eliminated all sub-OTUs with fewer than 10 reads overall. 43 fecal samples from 37 individuals were used for subsequent diet statistic analysis and 41 fecal samples from 35 individuals were used for gut microbiome analysis. The mean abundance of 16S and 18S data of multiple samples from same individuals was calculated.

### Statistical analysis

Sequence reads per fecal DNA sub-OTU found in the samples of each salamander were used as approximate proxy for ingested biomass. We calculated the prevalence of each prey taxon in the salamander diet, determined by the number of samples in which a prey category was found present or absent.

To evaluate alpha diversity (in-samples diversity) of salamander diets, OTU richness and Chao2 were calculated from prey prevalence data, and for bacteria ASV richness, Chao1 (estimated ASV richness) and Shannon indexes (estimated community diversity) were calculated from relative abundance data using the fossil package v 3.2.5^109^. To compare alpha diversity between location and sexes, a Kruskal Wallis One-Way Analysis of Variance was performed between locations and a Wilcoxon rank sum test was performed between sexes (due to non-normal distribution in both cases). To evaluate the differences of the prey prevalence data between different locations and sexes, Pearson’s Chi squared was calculated. Differences in microbial relative abundance between locations were assessed at the phylum, family and genus level, and differences between sexes were assessed at the phylum and family level, using the DESeq2 algorithm from the phyloseq package (v 1.30)^110^. Differentiallly abundant taxa were identified through applying the negative binomial Wald test with p-values corrected for multiple hypothesis testing using the Benjamini–Hochberg method^111^. The fire salamander gut microbiomes contained at least eight potentially pathogenic genera (*Flavobacterium*, *Chryseobacterium*, *Sphingobacterium*, *Aeromonas*, *Citrobacter*, *Yersinia*, *Acinetobacter*, *Stenotrophomonas*). The differences in relative abundances of pathogen load between locations were assessed using SPSS (IBM SPSS Statistics for Windows, Version 26.0. Armonk, NY, USA), with Kruskal–Wallis analysis followed by Dunn’s multiple comparison tests (significance values adjusted by the Bonferroni correction for multiple tests).

Redundancy analyses (RDA) were performed to examine the effect of location and sex on the relative abundance of ingested biomass, as well as to further quantify the effect of location, sex, SMI and diet on gut bacteria. Spearman’s Rank correlation was used to test whether the alpha diversity of individual diet and gut bacteria was significantly related to salamander SMI. Additionally, correlations were used to examine the relationship of alpha diversity of diet on gut bacteria.

To explore the beta diversity, principal coordinates analysis was performed, based on the Jaccard distance^112^, to compare community dissimilarities with presence or absence of OTUs for diet. We performed the principal coordinates analysis based on Bray–Curtis distance, to compare the community dissimilarities of relative abundance of ASVs, for both gut microbiome and pathogen load. We used PERMANOVA to test if the divergent species composition differed significantly between locations and sexes. Analyses were done with the package vegan (v 2.4)^113^ and the functions vegdist and adonis 2 in R (v 3.4).

To measure the correlation between individual salamander diet and the fecal microbiome, Mantel tests were performed on the Jaccard and Bray–Curtis distance matrices using R. To further explore the inter-relationship between the diet and the bacterial community matrices, a co-inertia analysis was run using the ade4 package^114^ in R.

## Supplementary Information

Supplementary Information.

## Data Availability

The demultiplexed raw amplicon reads were submitted to the Sequence Read Archive (NCBI-SRA) under Bioproject accession PRJNA768724 (18S data) and PRJNA767645 (16S data).
