# Supplementary material for: Diet diversity and environment determine the intestinal microbiome and bacterial pathogen load of fire salamanders
Source: Sci Rep. 2021 Oct 14;11:20493. doi: 10.1038/s41598-021-98995-6 (PMC8516891; doi:10.1038/s41598-021-98995-6)
Supplement: Supplementary file 1 — Supplementary Information. [file 41598_2021_98995_MOESM1_ESM.pdf]

Supplementary Information for

Diet diversity and environment determine the intestinal microbiome and bacterial pathogen load of fire salamanders

Yu Wang[1, +], Hannah K. Smith[1, +], Evy Goossens[2], Lionel Hertzog[3,4], Molly C. Bletz[5], Dries Bonte[3], Kris Verheyen[6], Luc Lens[3], Miguel Vences[5], Frank Pasmans[1] and An Martel[1, *]

Affiliations

[1] Wildlife Health Ghent, Department of Pathology, Bacteriology & Avian Diseases, Ghent University, Salisburylaan 133, 9820 Merelbeke, Belgium

[2] Department of Pathology, Bacteriology & Avian Diseases, Ghent University, Salisburylaan 133, 9820 Merelbeke, Belgium

[3] Terrestrial Ecology Unit (TEREC), Department of Biology, Ghent University, K. L. Ledeganckstraat 35, BE-9000 Ghent, Belgium

[4] Thünen Institute for Biodiversity, Bundesallee 68, 38116 Braunschweig, Germany

[5] Evolutionary Biology Lab, Zoological Institute, Braunschweig University of Technology, Mendelssohnstr. 4, 38106 Braunschweig, Germany

[6] Forest & Nature Lab, Department of Environment, Ghent University, Geraardsberge Steenweg 267, BE-9090 Gontrode, Belgium

*corresponding author: An.Martel@ugent.be (AM)

[+]these authors contributed equally to this work

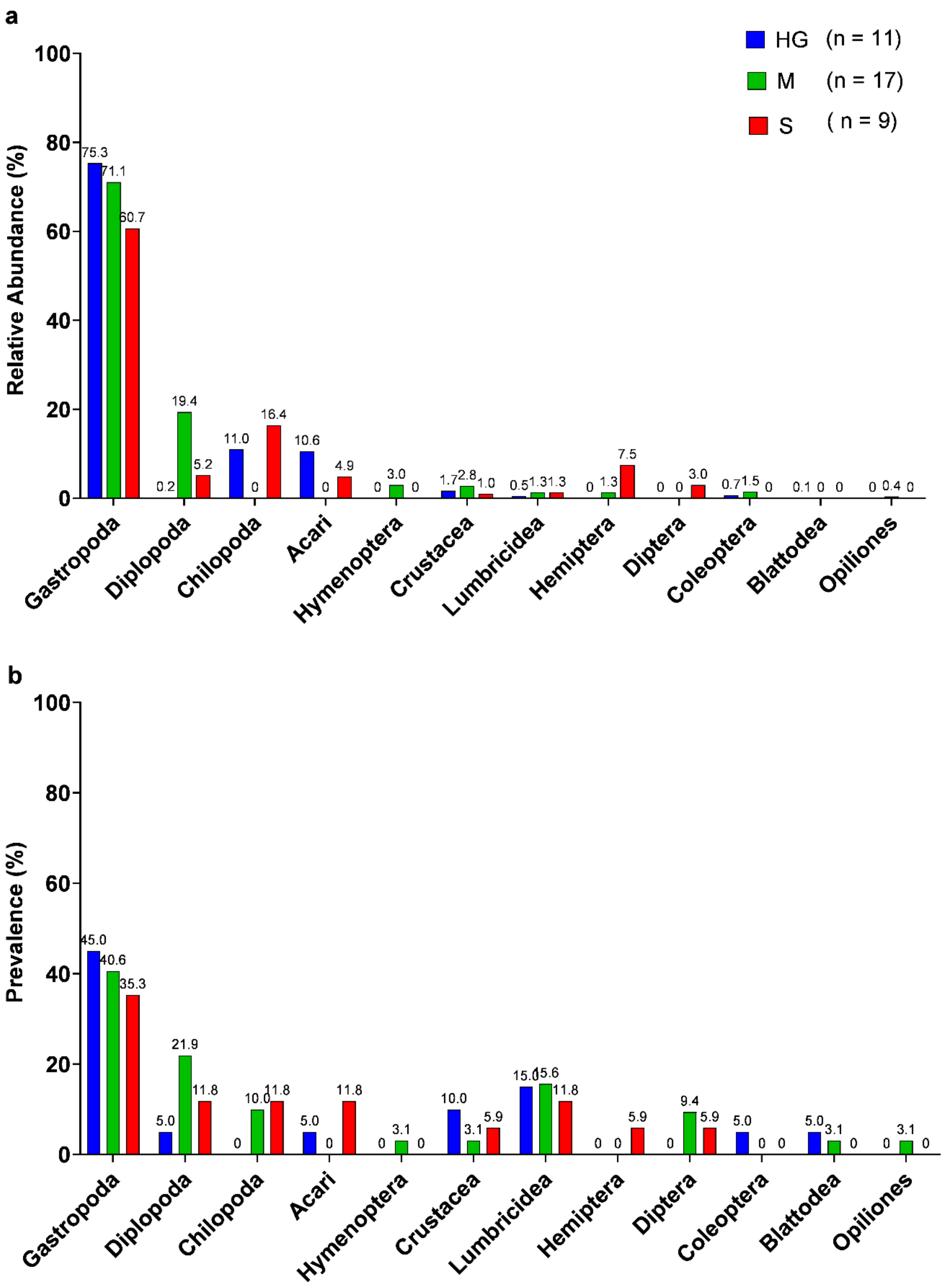

Supplementary Figure S1. Diet composition between forests. a). Relative abundance (relative number of sub-OTU reads for each prey) of fire salamander diet in forests HG, M, S. b). Prevalence of prey taxa (presence /absence) in fire salamander diet between forests HG, M, S. HG = Heilig Geestgoed, M = Makegem and S = Smetledebos.

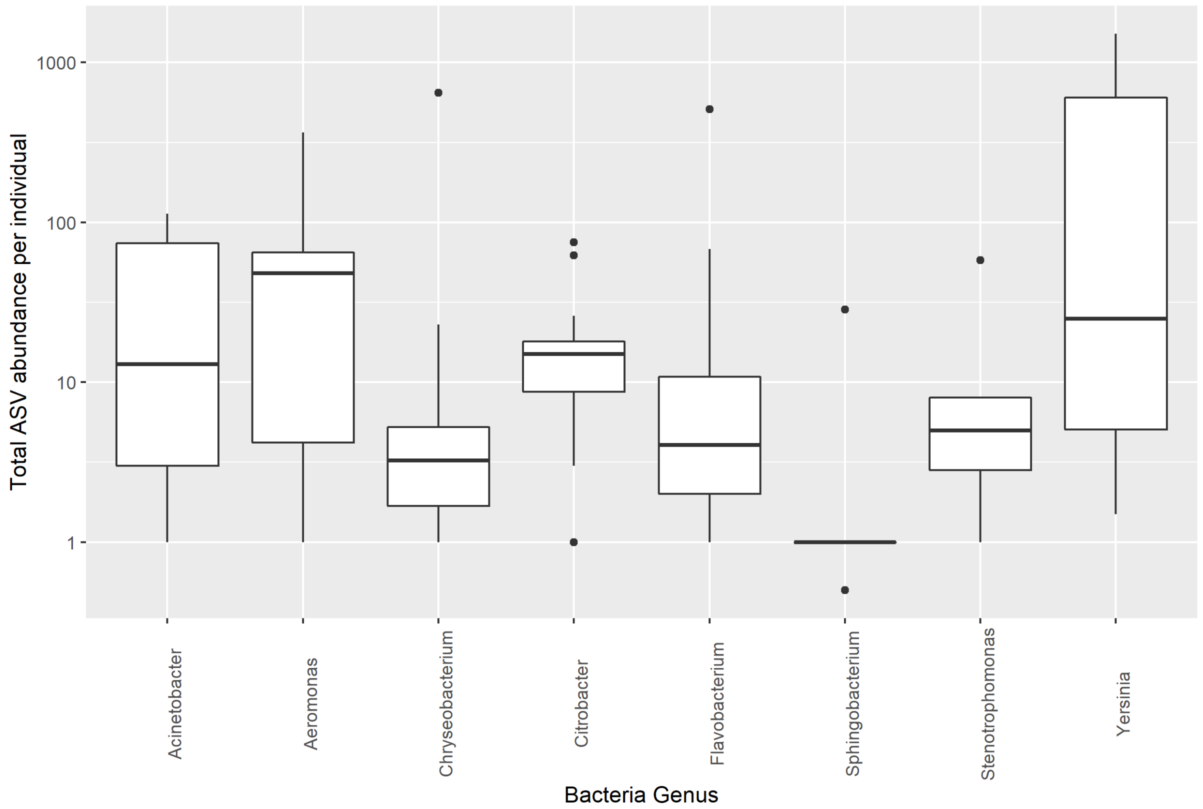

Supplementary Figure S2. Total ASV abundance per individual of each potential pathogenic bacteria in the fire salamander gut microbiome. Boxes are extended from the 25th to 75th percentiles, and the horizontal line inside the boxes defines the median. Whiskers indicate variability outside the upper and lower quartiles. Black circles indicate outliers.

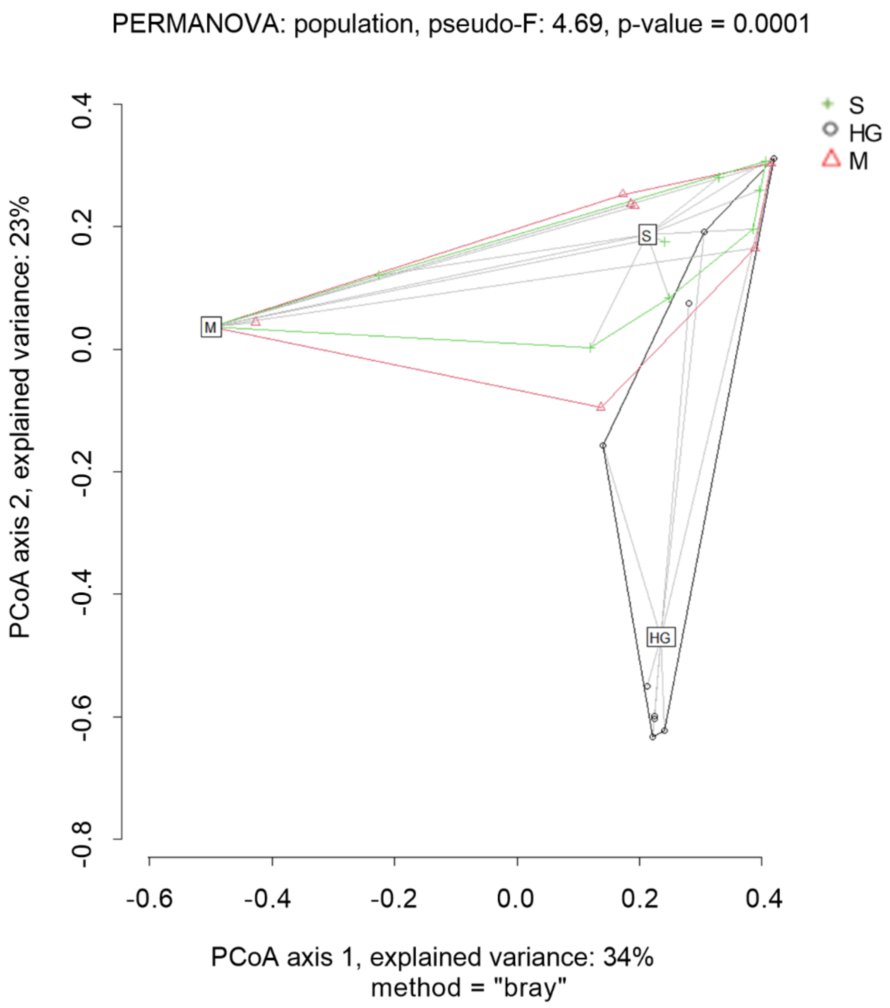

Supplementary Figure S3. Principal coordinates analysis of Bray-Curtis dissimilarity distance for total pathogen load of locations. HG = forest Heilig Geestgoed, M = forest Makegem and S = forest Smetledebos.

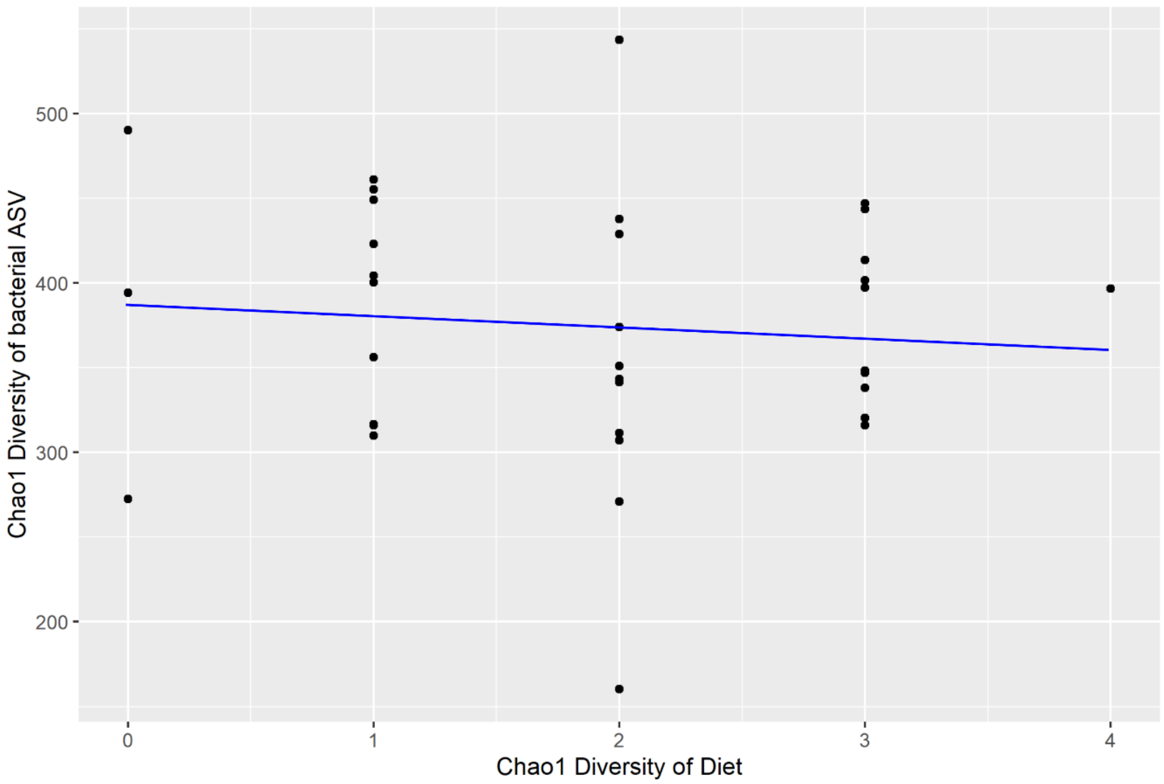

Supplementary Figure S4. Linear regression of the correlation between alpha diversity of fire salamander diet and gut microbiome.

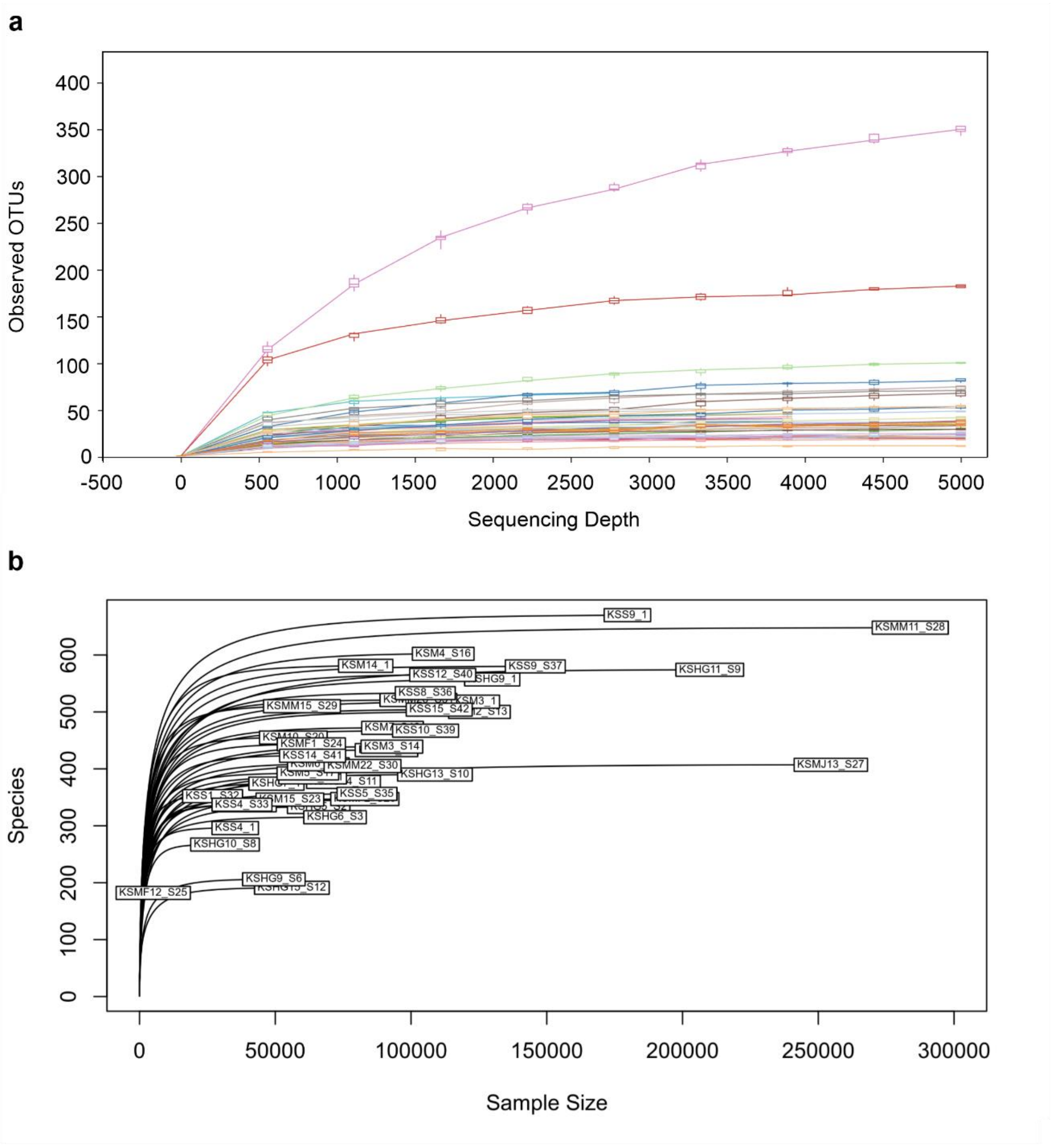

Supplementary Figure S5. Rarefaction curves of a) observed sub-OTU counts for 18S rRNA sequences, and b) the number of ASVs for 16S rRNA sequences in each sample. Each line represents one sample.

Phylum	Family	Mean relative abundance			p-value		
		HG	M	S	HG-M	HG-S	M-S
**Phylum level**							
Proteobacteria		25.6%	10.4%	12.6%	0.008	0.028	1
Elusimicrobia		0.5%	0.0%	0.0%	0.008	0.007	1
**Family level**							
Proteobacteria	*Yersiniaceae*	9.3%	0.2%	0.1%	< 0.001	< 0.001	1
Proteobacteria	*Aeromonadaceae*	0.9%	0.1%	0.0%	1	< 0.001	< 0.001
Elusimicrobiota	*Elusimicrobiaceae*	0.5%	0.01%	0.0%	0.137	0.045	1
Firmicutes	*Selenomonadaceae*	0.3%	1.1%	0.1%	1	0.922	0.025

Supplementary Table S1. Mean relative abundance and level of significance of bacterial phyla and families in fire salamander gut microbiome between different forests. HG = forest Heilig Geestgoed, M = forest Makegem and S = forest Smetledebos.

Phylum (sub)	Class (sub)	Order	Family	Species	F	BLAST
Mollusca	Gastropoda			*Alinda biplicata* (Two lipped door snail)	29	99.3
				Helicoidea sp. (land snail)	1	99.3
Annelida	Oligochatea		Lumbricidea	*Dendrobaena clujensis*	10	100
Anthropoda	Diplopoda			*Cylindroiulus punctatus (*Blunt-tailed snake millipede)	14	98
				Polydesmus sp.	6	98.6
				Proteroiulus fuscus	1	98.6
	Chilopoda			*Himantarium mediterraneum* (centipede)	3	97.3
	Arachnida	Opiliones		*Nelima sylvatica* (harvestman)	1	99.3
		Acari		*Acari* sp. (tick/mite)	5	96.6
				Atropacarus striculus (mite)	2	100
	Insecta	Hymenoptera		*Neodiprion* sp. (sawfly)	1	99.3
				Microplitis sp. (solitary endroparasitic wasp)	1	99.3
		Hemiptera		*Dactylopius coccus* (Cochineal scale insect)	1	94.7

		Diptera	*Haematobia irritans* (hornfly)	1	99.3
			Drosophila melanogaster (fruitfly)	1	99.3
			Bradysia hygida	1	97.3
			Ochlerotatus caspius	1	97.2
		Coleoptera	*Trochoideus goudoti* (fungus beetle)	1	99.3
		Blattodea	*Blattella germanica* (German cockroach)	2*	85.4
	Collembola		*Sminthurinus bimaculatus* (springtail)	8	97
Crustacea			*Peracarida* sp. (pillbugs/sowbugs)	1	95.3

Supplementary Table S2. Taxa found in fecal samples in functional taxonomic groups. Frequency of occurrence of each taxon. Species as given by the closest BLAST hit.
